# Resveratrol Promotes Nerve Regeneration via Activation of p300 Acetyltransferase-Mediated VEGF Signaling in a Rat Model of Sciatic Nerve Crush Injury

**DOI:** 10.3389/fnins.2018.00341

**Published:** 2018-05-23

**Authors:** Zhuofeng Ding, Jiawei Cao, Yu Shen, Yu Zou, Xin Yang, Wen Zhou, Qulian Guo, Changsheng Huang

**Affiliations:** Department of Anesthesiology, Xiangya Hospital of Central South University, Changsha, China

**Keywords:** resveratrol, nerve injury, nerve regeneration, p300, vascular endothelial growth factor

## Abstract

Peripheral nerve injuries are generally associated with incomplete restoration of motor function. The slow rate of nerve regeneration after injury may account for this. Although many benefits of resveratrol have been shown in the nervous system, it is not clear whether resveratrol could promote fast nerve regeneration and motor repair after peripheral nerve injury. This study showed that the motor deficits caused by sciatic nerve crush injury were alleviated by daily systematic resveratrol treatment within 10 days. Resveratrol increased the number of axons in the distal part of the injured nerve, indicating enhanced nerve regeneration. In the affected ventral spinal cord, resveratrol enhanced the expression of several vascular endothelial growth factor family proteins (VEGFs) and increased the phosphorylation of p300 through Akt signaling, indicating activation of p300 acetyltransferase. Inactivation of p300 acetyltransferase reversed the resveratrol-induced expression of VEGFs and motor repair in rats that had undergone sciatic nerve crush injury. The above results indicated that daily systematic resveratrol treatment promoted nerve regeneration and led to rapid motor repair. Resveratrol activated p300 acetyltransferase-mediated VEGF signaling in the affected ventral spinal cord, which may have thus contributed to the acceleration of nerve regeneration and motor repair.

## Introduction

Peripheral nerve injuries represent a significant source of patient morbidity and disability (Asplund et al., 2009; Kawamura et al., 2010). Although axons in peripheral nerves have the capacity to regenerate after injury, a number of clinical reports and studies in recent years have indicated that functional recovery, especially motor function, is far from satisfactory even with advances in surgical procedures (Ruijs et al., 2005; Grinsell and Keating, 2014; Sakuma et al., 2016). The prevalent hypothesis for this is that the injury-induced increase in axonal growth is too slow and that, consequently, by the time axons reach distal denervated nerves, the milieu has become non-permissive for growth (Sulaiman and Gordon, 2000; Gordon et al., 2011). This long delay for axons to reach their targets is thought to prevent functional restoration (Sakuma et al., 2016).

A classical way to accelerate growth in peripheral axons is to activate their intrinsic growth programs, which consist of the expression of a number of signaling factors (Zigmond, 2011; Struebing et al., 2017). The family of vascular endothelial growth factors (VEGFs, including VEGFa, VEGFb, and their receptors VEGFR1, VEGFR2, and neuropilin-1) has been implicated as a potent mediator of nerve regeneration (Carmeliet and Storkebaum, 2002; Mackenzie and Ruhrberg, 2012; Carmeliet and de Almodovar, 2013; Licht and Keshet, 2013; Guaiquil et al., 2014). *In vivo*, VEGFs are expressed after peripheral nerve injury (Li et al., 2012; Carmeliet and de Almodovar, 2013). Rodents lacking VEGFs showed impaired nerve regeneration (Sun et al., 2004; Li et al., 2008; Yu et al., 2008). The treatment of VEGFa or VEGFb induced extensive neurite growth and mediated nerve repair (Mackenzie and Ruhrberg, 2012; Guaiquil et al., 2014). Both VEGFa and VEGFb can exert this effect on nerve regeneration independent of their angiogenic roles. In fact, VEGFa and VEGFb, by signaling via their receptors, have direct effects in neuron cell bodies and promote axon elongation and nerve regeneration after peripheral nerve injury (Carmeliet and Storkebaum, 2002; Guaiquil et al., 2014).

Resveratrol (Res), a polyphenolic compound naturally present in foods, is widely known for its benefits in controlling multiple targets involved in oxidative stress, inflammation, and cell death (Pervaiz and Holme, 2009). The VEGF family is one of the targets by which Res exerts its beneficial effects on the nervous system. In two independent studies, Hermann et al. (2015) and Dong et al. (2008) found that Res increased the level of VEGFs in the injured brain and thus might attenuate brain damage. However, Res may show a contrasting effect on the same target in different organs or cells. For example, Res has been shown to reduce VEGFs in endothelial cells and tumor cells (Trapp et al., 2010). Res produces this therapeutic effect by suppressing the Akt and ERK pathways in tumor cells (Jiang et al., 2009; Banerjee Mustafi et al., 2010). Conversely, Res produces neuroprotective effects by activating Akt signaling in the nervous system (Zhou et al., 2014; Liu et al., 2015; Yin et al., 2017). Therefore, under certain pathophysiological conditions it is necessary to identify the cellular targets responsible for the effects of Res to develop target-specific therapies. This will be helpful in increasing the efficacy of this drug without increasing its potential adverse effects (Chung et al., 2012).

Res has been shown to have neuroprotective effects in the treatment of various types of injuries of the central nervous system (CNS), including stroke, traumatic brain injury, subarachnoid hemorrhage, and spinal cord injury (Singleton et al., 2010; Lopez et al., 2015). Many studies including our previous work have attributed the beneficial role of Res to the activation of various cellular targets, such as silent mating type information regulation 2 homolog 1 (Sirt1) (Borra et al., 2005; Leheste and Torres, 2015), AMP-activated kinase (Dasgupta and Milbrandt, 2007) and nuclear factor-like 2 (Chen et al., 2005). In addition, Res has also shown protective effects in the treatment of animal models of peripheral nerve injury. For example, Kim et al. found that Res increased the survival of ganglion cells after optic nerve transection (Kim et al., 2013). Lindsey et al. showed protection of injured retinal ganglion cell dendrites after long-term dietary Res administration (Lindsey et al., 2015). Bagriyanik et al. found that Res ameliorated myelin and axon damage caused by chronic constriction injury of the sciatic nerve (Bagriyanik et al., 2014). However, the mechanism underlying the protective effect of Res has not been well-clarified in the peripheral nervous system. In particular, it is not clear whether Res could act on an intrinsic factor to promote neurite growth and axon elongation, especially within a short treatment period after the peripheral nerve injury.

In this study, we hypothesized that Res could upregulate the expression of VEGFs and mediate nerve regeneration and motor repair in a rat model of sciatic nerve crush injury. Res treatment was given immediately after the nerve injury and repeated once daily for 10 consecutive days. The histopathological and behavioral outcomes were examined. The role of VEGFs in the Res-induced nerve regeneration was investigated. In addition, we also explored the upstream signaling that regulates the expression of VEGFs after Res treatment.

## Materials and methods

### Animal and drugs

Experiments were carried out in adult Sprague Dawley rats (male, 200–220 g) purchased from Hunan SLAC Laboratory Animal Co., LTD. Three rats were housed per cage in a temperature-controlled (25–28°C) and pathogen-free room on a 12-h light/dark cycle and with free access to autoclaved food and water. Behavioral experiments were conducted between 8:00 am and 4:00 pm. All protocols were approved by the Animal Care Committee of Central South University. All efforts were made to minimize the animals' suffering.

Res was obtained from Sigma-Aldrich (Cat V900386; St. Louis, MO, USA). It was diluted to a concentration of 50 mg/ml in 2%V/V DMSO saline solution to make a suspension for further administration. C646 (Cat S7152) and Axitinib (Cat S1005) were obtained from Selleck Chemicals (Shanghai, CN). LY294002 was obtained from Cell Signaling Technology (Cat #9901s; Danvers, MA, USA). DMSO was obtained from MP Biomedicals (Cat 02191418; CA, USA).

### Sciatic nerve crush

Sciatic nerve crush was performed according to our previous report (Zou et al., 2016). The crush lesion was induced by a hemostat forceps. The applied force was 1,000 g at the tip of the forceps, which were calibrated by a force-sensing resistor (FSR400, Interlink Electronics, CA, USA) linked to an Avometer. Animals were anesthetized with isoflurane; the left sciatic nerve was exposed at high-thigh level under aseptic conditions and then crushed with a fine hemostat at a cycle of 10 s clamping/5 s loosening three times. The wound was then closed with two to three skin sutures (size 3–0). In sham-operated animals, the nerve was isolated and manipulated without inducing crush injury.

### Drug administration

Res was administered intraperitoneally (i.p.) at a dose of 50 mg/kg (*n* = 12) or 200 mg/kg (*n* = 12), according to previous reports (Ates et al., 2006; Liu et al., 2011). The treatment was performed once daily for 10 days after the crush injury. Sham-operated rats received the vehicle (2%V/V DMSO in saline solution) as a control (*n* = 12). For rats that were assigned to receive C646 (an antagonist of p300 acetyltransferase) and Axitinib (an inhibitor of VEGF signaling), an intrathecal catheter was placed before the crush injury. The intrathecal catheter was placed according to our previous study (Ding et al., 2017b). Briefly, the bony arch of the vertebra was exposed between the L4 and L5 vertebrae. A PE-10 catheter was then inserted into the subarachnoid space until it reached the lumber enlargement. A successful intrathecal catheter placement was confirmed by a lidocaine test. For rats assigned to receive C646 and Axitinib treatment, induction of sciatic nerve crush injury was only performed in those with successful intrathecal catheter placement. C646 and Axitinib were delivered intrathecally once daily for 10 days at a concentration of 10 μM diluted in a total volume of 10 μl 1%V/V DMSO in saline solution (*n* = 8 for each treatment). Rats that received the vehicle (10 μl 1%V/V DMSO in saline solution) were used as controls (*n* = 8).

### Behavioral tests

#### Sciatic function index

The sciatic function index (SFI) was calculated from walking track analysis by recording rats' footprints using equipment which contains an alley (5 × 7.5 × 42 cm) ending in a darkened cage (Fey et al., 2010). Before recording, all rats were allowed to explore the alley three times as conditioning trials. After practice, they walked straight to the darkened cage. The floor of the alley was then covered with white paper and the facies volaris of the posterior limbs of the rat were smeared with stamp-pad ink. The rats were placed at the entrance of the corridor; they then walked along the alley leaving footprints on the white paper. The tests continued until five measurable footprints were recorded. From the footprints, several parameters were measured: (i) print length (PL; distance from the top of the third toe to the heel); (ii) toe spread (TS; distance between the first and the fifth toe), and (iii) intermediary toe spread (IT; distance from the second to the fourth toe). The three measures were taken both from the normal foot (NPL, NTS, and NIT) and from the operated or experimented foot (EPL, ETS, and EIT). SFI was calculated using the following formula: SFI = −38.3 × PLF + 109.5 × TSF + 13.3 × ITF−8.8, in which (i) print length factor (PLF) = (EPL−NPL)/NPL; (ii) toe spread factor (TSF) = (ETS−NTS)/NTS; (iii) intermediary toe spread factor (ITF) = (EIT−NIT)/NIT. In normal rats the SFI score is 0, while a score of 100 indicates total impairment.

#### Toe spreading distance

The extent of spontaneous toe spreading distance from the first to the fifth toe was measured when the rats were held in a vertical position which caused them to spread their toes reflexively. Toe spreading was normalized by subtracting the spreading distance of the normal paw from that of the operated paw (Kanaya et al., 1996; Bervar, 2000).

#### Open field locomotor performance

Rats were placed in an open field test box (100 × 100 × 40 cm) and allowed to explore the field for 5 min to analyze locomotor function. Locomotor performance was evaluated according to a scale adapted from Basso et al. (1995). Possible scores ranged from 0 (total paralysis) to 14 (normal gait). Detailed score rating scales are listed in Table 1.

**Table 1:** Scales for open field locomotor performance.

**Score**	Status of operated limb^*^
0	Paralysis
1	Limb dragging
2	Plantar placement without weight support
3	Plantar placement with weight support
4	*Occasional* plantar stepping and *frequent* or continuous heel placement
5	*Occasional* plantar stepping and *occasional* heel placement
6	*Frequent* plantar stepping and *frequent* or *continuous* heel placement
7	*Frequent* plantar stepping and *occasional* heel placement
8	*Continuous* plantar stepping and *frequent* or *continuous* heel placement
9	*Continuous* plantar stepping and *occasional* heel placement
10	Toe clearance and continuous plantar stepping without heel placement
11	Parallel placement or toe spreading and toe clearance and *continuous* plantar stepping without heel placement
12	Parallel placement *and* toe spreading and toe clearance and *continuous* plantar stepping without heel placement
13	Heel of the ground in stance and parallel placement *and* toe spreading and toe clearance and *continuous* plantar stepping without heel placement
14	Normal gait

* *Occasional: < 50%,Frequent: 51–94%, Continuous: ≥95% of the observation time*.

### Tissue harvesting

All rats were deeply anesthetized with isoflurane. Four rats from each group were used for immunofluorescence and toluidine blue staining. After perfusion with ice-cold PBS followed by 4% paraformaldehyde, the lumbar section of the spinal cords and the sciatic nerve were dissected out and post-fixed at 4°C overnight. The distal part of the sciatic nerves 0.5 cm from the operative sites was cut for immunofluorescence and toluidine blue staining. Eight rats from each group were used for western blot and PCR analyzation. Rats were decapitated and lain in ice bags; the ventral spinal cords were quickly removed and stored at −80°C for further testing.

### Cell culture and intervention

Differentiated PC-12 cells were ordered from the type culture collection of the Chinese academy of sciences Shanghai, China. Cells were cultured in RPMI-1640 medium (10% fetal bovine serum, 100 U penicillin/ml and 100 mg streptomycin/ml). For drug intervention, Res and LY294002 were diluted in 25 μM with complete medium. C646 and Axitinib were added at a final concentration of 10 μM, with 0.1% V/V DMSO given as a vehicle control. For siRNA transfection, siRNAs were diluted to a concentration of 100 nM and transfected to cultured cells by riboFECT™ CP buffer (Cat C710511-05; Guangzhou, CN), according to the manufacturer's instructions. Three candidate siRNAs were designed: si-P300-1, CCAAATAACCTTTCTCCAT; si-P300-2, GCAAGCAGTCATCTATTTA; si-P300-3, GCATGCATGTTCAAGAATA. The siRNA-transfected cells were collected 1 or 2 days later for PCR or western blot and immunostaining analysis.

### Real-time quantitative PCR

Total RNA collected from ventral horn of the lumbar spinal cord or cultured cells was extracted using an E.Z.N.A. Total RNA Kit (Omega, China). RNA concentration was assessed by spectrophotometry at 260 and 280 nm absorbance (NanoDrop ND-2000 Spectrophotometer; Thermo Fisher Scientific). First-strand cDNA was synthesized using the PrimeScript RT reagent Kit (Takara, Japan). Real-time quantitative PCR (RT-qPCR) was conducted with a Quantifast SYBR Green PCR Kit purchased from GeneScript (China). A volume of 20 μL/well reaction system was used which contained the following reagents: 10 μL Quantifast SYBR Green PCR Master Mix (2 ×), 1 μL forward primer, 1 μL reverse primer, 1 μL cDNA and 2 μL RNase-free water. The cycling conditions were set as the following: PCR intimal heat activation at 95°C for 5 min, followed by 40 cycles at 95°C for 10 s, 60°C for 25 s and extension at 72°C for 25 s. The melt curve stage was set as 95°C for 5 s, one cycle at 60°C for 1 min and one cycle at 50°C for 30 s. Relative quantities of the candidate genes and β-actin were calculated using the previously described comparative threshold cycle (ΔΔCt) method (Xu et al., 2016). Primers for RT-qPCR are listed in Table 2.

**Table 2:** Primers for real-time quantitative polymerase chain reaction.

**Gene**		**Primer**
β-actin	Forward	TCGTGCGTGACATTAAAGAG
	Reverse	ATTGCCGATAGTGATGACCT
Rab13	Forward	CTACCGCCTATTACCGTGGA
GAP43	Reverse	TCACACTTGTTTCCCAGCAG
	Forward	CAGGAAAGATCCCAAGTCCA
	Reverse	GAACGGAACATTGCACACAC
bcl-2	Forward	CTTCAGGGATGGGGTGAACT
	Reverse	CAGCCTCCGTTATCCTGGAT
VEGFa	Forward	GCCCATGAAGTGGTGAAGTT
	Reverse	ACTCCAGGGCTTCATCATTG
VEGFb	Forward	TTTTTCCACGGGCTTTACAC
	Reverse	TGCATTTCCTTTTTGGAACC
VEGFR1	Forward	AAAGTCCGTGTCGTCCCTTC
	Reverse	ACAGCCACTTGATGGTAGGC
VEGFR2	Forward	AGATGCGGGAAACTACACGG
	Reverse	GGGAGGGTTGGCATAGACTG
MnSOD	Forward	ATCTGAACGTCACCGAGGAG
	Reverse	TAGGGCTCAGGTTTGTCCAG
Sirt1	Forward	CGAGTGCTTCGCAGGATTTG
	Reverse	CCCTCAATCTGTTGACGGCT

### Toluidine blue staining

After being deeply anesthetized, rats were perfused with ice-cold PBS followed by 4% paraformaldehyde. The sciatic nerve 0.5 cm distal to the lesion site was dissected and post-fixed at 4°C overnight. Fixed sciatic nerves were embedded in Epson 812 and cut into semi-thin (500 nm) sections. The sections were stained with 1% toluidine blue and photos were acquired using a light microscope (Leica, Germany).

### Immunohistochemistry

After being blocked for 1 h at 37°C in PBS containing 0.5% donkey serum and 0.3% Triton X-100, frozen sections of fixed tissues or cell creep plate were incubated with primary antibody at 4°C overnight. The following primary antibodies were used: rabbit anti-VEGFR1 (1:100, Abcam), rabbit anti-VEGFR2 (1:100, Proteintech), mouse anti-NeuN (1:400, Novus), mouse anti-p300 (1:100, Santa Cruz), rabbit anti-Phospho-P300 pSer1834 (1:200, Invitrogen), rabbit anti-NF200 (1:100, Sigma), and rabbit anti-MAP2(1:400, Abcam). For fluorescence immunostaining, species-specific donkey antibodies conjugated to Alex Fluor 488 or 594 (1:400, Jackson Lab) were applied. Following immunostaining procedures, images were captured from three immunohistochemical sections of each sample using a fluorescence microscope (Leica, DM 5000B, Germany) and analyzed with Image Pro Plus 6.0.

### Quantification of the neurite length in differentiated PC-12 cells

Differentiated PC-12 cells (type culture collection of the Chinese academy of sciences Shanghai, China) were cultured at a density of 10^4^ cells/mL in a 6-well plate containing collagen IV coated glass cover slips (Thermo Fisher Scientific). Cells were grown in the presence or absence of 25 μM Res or the combinations of Res and 10 μM Axitinib for 24 h. The immunohistochemistry method was used as described above. The neurite of differentiated PC-12 cells was identified by the MAP2-positive staining. The distance from the top of neurite to the edge of nucleus was measured using Image Pro Plus 6.0 software. Eight fields in each group were randomly selected for the analyzation. The length of neurite was averaged from the total length of observable neurites per cell.

### Western blot

The ventral lumbar spinal cords were isolated rapidly and immediately snap frozen on dry ice. Frozen tissue was homogenized in RIPA lysis buffer (1% Triton X-100, 1% deoxycholate, 0.1% SDS, 20 mM Tris, 0.16 M NaCl, 1 mM EDTA, 15 mM NaF, 1 mM EGTA, and 4 mM Na3VO4), supplemented with a protease inhibitor cocktail. Total protein was isolated after centrifugation at 4°C for 15 min at 1,000 g. The samples from the cultured differentiate PC-12 cells were directly dispersed in lysis buffer, centrifuged and sonicated. Protein samples (of total protein) were diluted in SDS sample buffer and heated at 99°C for 10 min followed by measurement of protein concentration by BCA assay (Thermo Fisher Scientific). A total of 50 μg denatured protein sample per lane was loaded onto an 8% SDS-polyacrylamide gel (Bio-Rad Laboratories) for electrophoresis and then transferred onto a polyvinylidene difluoride membrane (Minipore, Hercules, USA). After being blocked with 5% non-fat milk in Tris-buffered saline containing 0.1% Tween 20 for 2 h, the membranes were then incubated at 4°C overnight with the following primary antibodies: rabbit anti-Phospho-P300 (1:500, Invitrogen), mouse anti-p300 (1:200, Santa Cruz Biotechnology), rabbit anti-pAkt (1:500, Cell Signaling Technology), mouse anti-H3K27ac (1:500, Active Motif),rabbit anti-Akt (1:500, Cell Signaling Technology), rabbit anti-actin (1:1000, Sigma), and rabbit anti-VEGFb (1:200, Santa Cruz Biotechnology). The proteins were detected by HRP-conjugated anti-rabbit or anti-mouse secondary antibody (1:5000, Cell Signaling Technology) and visualized using an ECL Plus blot kit (Minipore, Hercules, USA). Images were acquired using a ChemiDoc XRS System with Image Lab software (Bio-Rad Laboratories). Band intensities were quantified with densitometry using Image Lab software (Bio-Rad Laboratories). Band intensities of the target proteins were normalized to that of actin.

### Statistical analysis

All raw data are presented as mean ± SEM and were statistically analyzed using one-way or two-way analysis of variance with repeated measures followed by Bonferroni *post hoc* tests. *P*-values < 0.05 were considered statistically significant.

## Results

### Res dose-dependently improved motor function and nerve regeneration after crush injury

**Figure 1:**
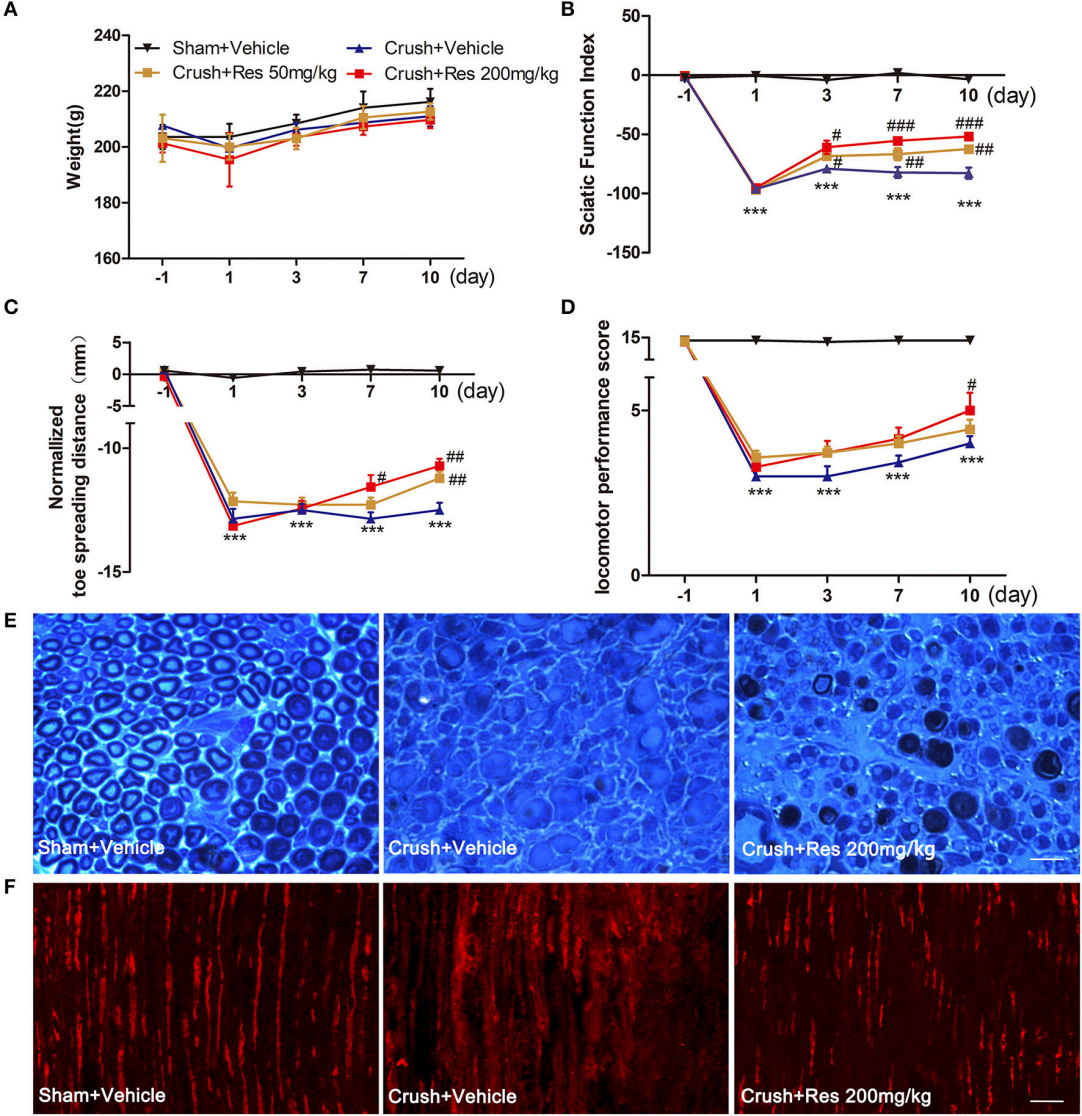
Resveratrol (Res) dose-dependently improved motor function and nerve regeneration after crush injury. **(A)** Rats did not lose weight either after the sciatic nerve crush injury or after the daily intraperitoneal administration of Res at a dose of 50 mg/kg or 200 mg/kg for 10 consecutive days. **(B–D)** The sciatic function index, normalized toe spreading distance and locomotor performance score were significantly decreased from the first day to 10 days after crush injury (compared Crush + Vehicle with Sham + Vehicle, ^***^P<*0.001*. *n* = 12 for each group). The sciatic nerve index was increased by the both doses of Res from the third to the tenth days after the treatment. The normalized toe spreading distance was increased by 50 mg/kg and 200 mg/kg Res on the tenth day and from the seventh to tenth days after the treatment, respectively. The locomotor performance score was increased by 200 mg/kg Res on the tenth day after the treatment (compared Crush + 50 mg/kg Res or Crush + 200 mg/kg Res with Crush + Vehicle, ^#^*P*<*0.05*, ^##^*P*<*0.01*, ^*###*^*P*<*0.001*. *n* = 12 for each group). **(E)** Representative images of toluidine blue staining showed the degeneration and regeneration of myelinated axons in the crush injury rats receiving the vehicle and Res treatment, respectively. The transverse sections were obtained from the distal part of the injured nerve. Scale bar = 50 μm. **(F)** Representative images of immunofluorescence staining showed the degeneration and regeneration of NF200 positive axons (red) in the crush injury rats receiving the vehicle and Res treatment, respectively. The longitudinal sections were obtained from the distal part of the injured nerve. Scale bar = 50 μm.

Within the treatment period, rats that had received sciatic nerve crush injury did not show any reduction in body weight when compared with the sham-operated rats (Figure 1A), indicating that the treatment is not likely to cause toxicity (Johnson et al., 2011). The crush injury resulted in motor deficit in the affected limb, demonstrated by dramatic decreases in SFI, normalized toe spreading distance and locomotor performance score. Res treatment at both 50 and 200 mg/kg increased SFI as early as 3 days after the treatment (*P* < 0.05), although the improvement was most marked at the higher dose (Figure 1B). Similarly, Res alleviated the adduction of the injured paw, leading to increased normalized toe spreading distance from 7 days after the 200 mg/kg Res treatment (*P* < 0.05) and 10 days after the 50 mg/kg treatment (*P* < 0.01). In addition, the normalized toe spreading distance was more improved after the 200 mg/kg Res treatment than after the 50 mg/kg Res treatment (Figure 1C). Res increased locomotor performance score at 10 days after the treatment. However, the improvement was observed only in rats receiving the 200 mg/kg dose (*P* < 0.05; Figure 1D). Among the three parameters, SFI was the most sensitive to reflect the alleviation of motor deficit, as the increase of SFI was observed as early as 3 days after the treatment, much earlier than the increase of the other two parameters. These results indicate that Res promoted motor repair in a dose dependent manner after the sciatic nerve crush injury. Since Res treatment at 200 mg/kg led to a better and faster degree of motor repair, further experiments were performed at this dose to investigate the effect and mechanism of Res-promoted nerve regeneration.

The motor component of the sciatic nerve is mainly composed of the large myelinated axons efferent from the neurons in the ventral spinal cord. Toluidine blue staining showed that most of the myelinated axons in the distal part of the lesion site were degenerated after the crush injury. After Res treatment for 10 days, myelinated axons grew into the distal part of the lesioned nerve. The newly generated axons manifested either as axons with a thin epineurium or as axonal fasciculation. However, these manifestations of nerve regeneration were only sparsely identified in the sciatic nerve of rats receiving vehicle treatment (Figure 1E). NF200 is a marker of large myelinated axons. The number of NF200-positive axons in the sciatic nerve 0.5 cm distal to the lesion site was dramatically decreased 10 days after the crush injury. However, there were markedly more NF200-positive axons in rats treated with Res than those treated with the vehicle (Figure 1F). These results may indicate that Res promoted motor nerve regeneration after the sciatic nerve crush injury.

### Activation of VEGF signaling may contribute to the res-induced nerve regeneration

**Figure 2:**
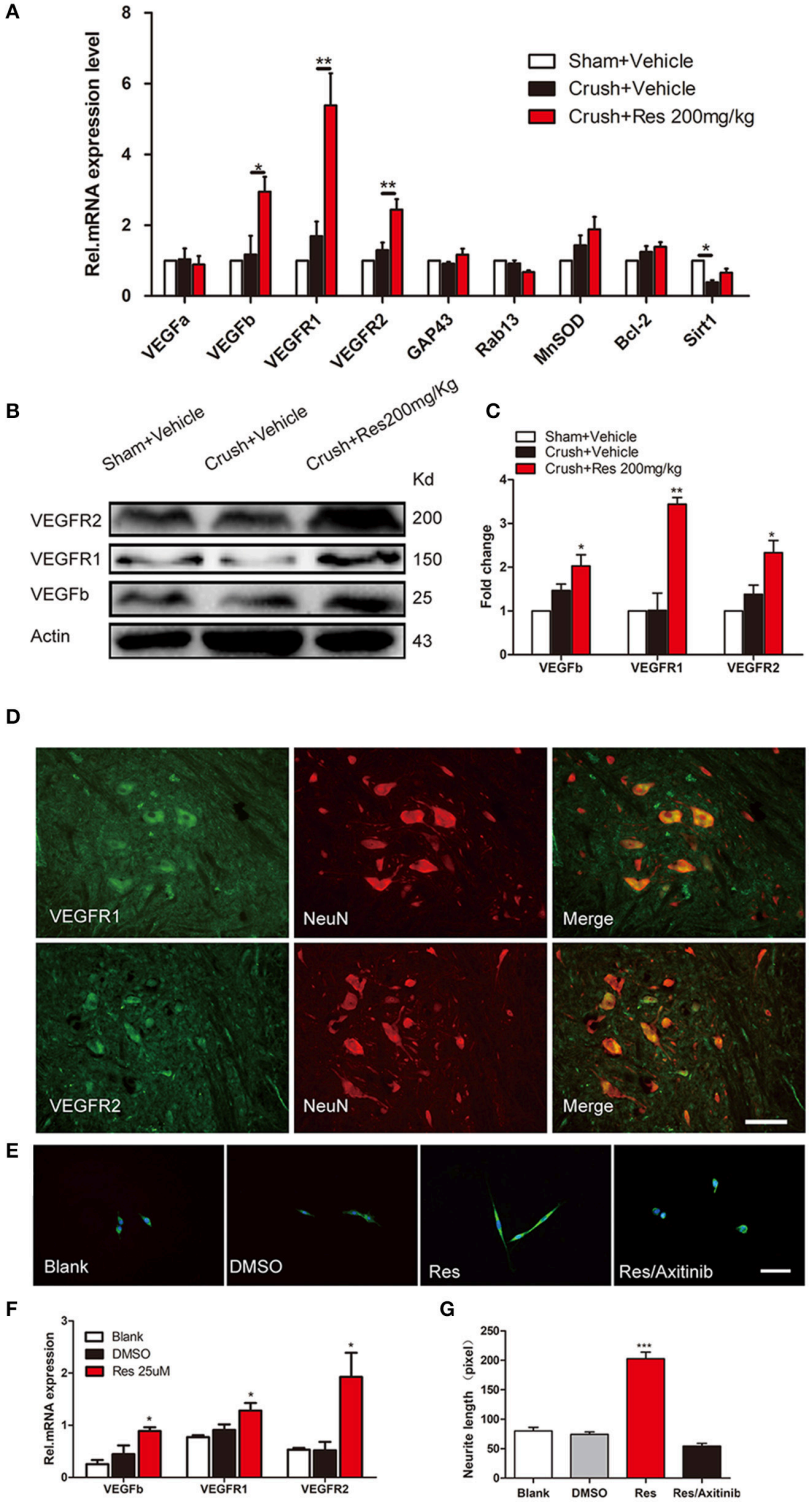
Resveratrol (Res) up-regulated the expression of VEGFs in rat ventral spinal cord **(A–D)** and the inhibition of VEGFs suppressed the Res-induced axon growth in differentiated PC-12 cells **(E–G)**. **(A)** The crush injury reduced the mRNA expression of Sirt1 but did not affect the expression of other genes. Res (200 mg/Kg) increased the mRNA expression of VEGFb, VEGFR1 and VEGFR2 but did not alter the expression of other genes in rat ventral spinal cord (compared with Crush + Vehicle, ^*^P<*0.05*, ^**^P<*0.01, n* = 8 for each group). **(B,C)** Res (200 mg/Kg) increased the protein expression of VEGFb, VEGFR1, and VEGFR2 in rat ventral spinal cord (compared with Crush + Vehicle, ^*^P < 0.05,^**^P < 0.01. n = 8 for each group). **(D)** VEGFR1 and VEGFR2 were mostly co-labeled with NeuN, a neuronal marker, in rat ventral spinal cord. Scale bar = 50 μm. **(E)** Representative images of MAP2 (green) and DAPI (blue) staining showed the neurite length of differentiated PC-12 cells exposed to DMSO, Res and Res plus Axitinib (an inhibitor of VEGF signaling), respectively. Scale bar = 50 μm. **(F)** Res enhanced the relative mRNA expression of VEGFb, VEGFR1, and VEGFR2 in differentiated PC-12 cells (compared with DMSO treated cells, ^*^P<*0.05*). **(G)** The inhibition of VEGF signaling by Axitinib suppressed the Res-induced axon growth in differentiated PC-12 cells (compared with Res treated cells, ^***^P<*0.001*).

The mRNA expression of a number of potential targets of Res was examined in the ventral spinal cord where the affected motor neurons were located. These target genes have been associated with Res-induced neuroprotective and nerve regeneration effects, and include VEGFa, VEGFb, VEGFR1, VEGFR2, GAP43, Rab13, MnSOD, Bcl-2, and Sirt1. The mRNA expression of Sirt1 was decreased after the crush injury when compared with sham (*P* < 0.05). Although a trend of increased Sirt1 expression was observed after Res treatment, it did not reach the same level as the sham. The mRNA expression of GAP43, RAB13, MNSOD, and Bcl-2 was not changed, either by the crush injury or by Res treatment. The mRNA expression of VEGFa, VEGFb, VEGFR1, and VEGFR2 was also not altered by the crush injury. However, Res significantly up-regulated the mRNA expression of VEGFb (*P* < 0.05), VEGFR1 (*P* < 0.01) and VEGFR2 (*P* < 0.01; Figure 2A). Consistent with this, the protein levels of VEGFb (*P* < 0.05), VEGFR1 (*P* < 0.01), and VEGFR2 (*P* < 0.05) were also increased after Res treatment (Figures 2B,C). The expression of VEGFR1 and VEGFR2 was mostly localized in the motor neurons within the ventral spinal cord (Figure 2D). Therefore, we believe that the enhanced expression of VEGFb may act on VEGFR1 and VEGFR2 and thus facilitate signaling transduction in the spinal motor neuron. In PC-12 cells, Res up-regulated the expression of VEGFb, VEGFR1, and VEGFR2, as well as promoting neurite elongation. The inhibition of VEGF signaling by Axitinib suppressed the Res-induced neurite growth (Figures 2E–G). These results indicate that the activation of VEGF signaling contributed to the Res-induced nerve regeneration.

### Res enhanced Akt phosphorylation of p300 at Ser-1834

**Figure 3:**
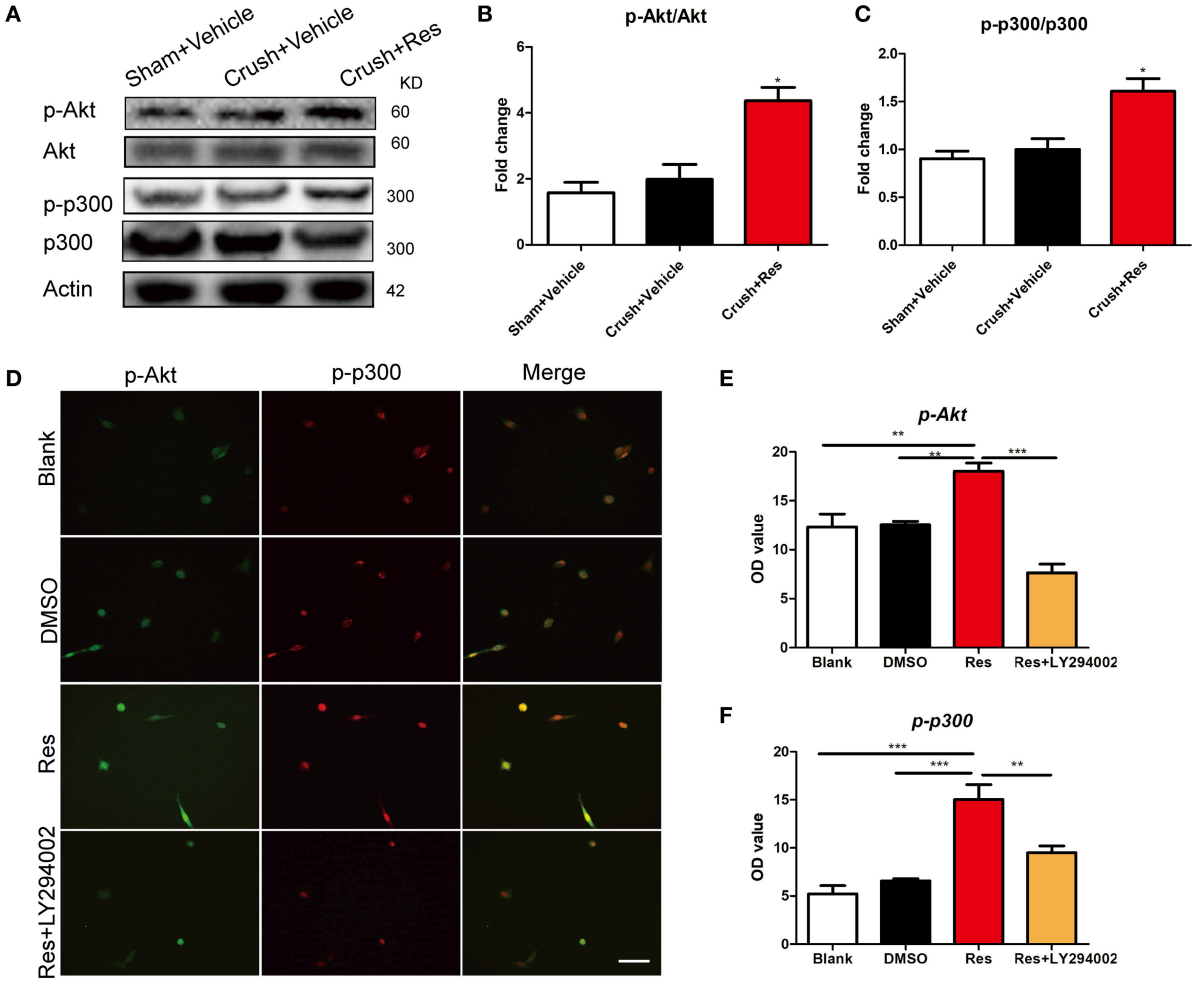
Resveratrol (Res) enhanced the phosphorylation of Akt (p-Akt) and p300 (p-p300) in rat ventral spinal cord **(A–C)** and the inhibition of p-Akt suppressed the Res-induced expression of p-p300 in differentiated PC-12 cells **(D–F)**. **(A)** The representative protein bands showed the expression of p-Akt, Akt, p-p300 and p300 in rat ventral spinal cord. **(B)** Res (200 mg/Kg) increased the ratio of p-Akt to Akt in rat ventral spinal cord (Crush + Res vs. Crush + Vehicle, ^*^P<*0.05*. *n* = 8 for each group). **(C)** Res (200 mg/Kg) increased the ratio of p-p300 to p300 in rat ventral spinal cord (Crush + Res vs. Crush + Vehicle, ^*^P < 0.05. *n* = 8 for each group). **(D)** Representative images showing the co-labeling of p-Akt and p-p300 in differentiated PC-12 cells. Scale bar = 50 μm. **(E)** LY294002, an inhibitor of Akt signaling, suppressed the Res-induced immuno intensity of p-Akt in differentiated PC-12 cells. (compared with Res treated cells, ^**^P<*0.01*, ^***^P<*0.01*). **(F)** LY294002 suppressed the Res-induced immuno intensity of p-p300 in differentiated PC-12 cells. (compared with Res treated cells, ^**^P<*0.01*, ^***^P<*0.01*).

In rats subjected to sciatic nerve crush injury, Res treatment increased the phosphorylation of Akt (p-Akt) as well as the phosphorylation of p300 at Ser-1834 (p-p300) in the ventral spinal cord (*p*<*0.05*). However, Res did not alter the overall expression of Akt and p300. There were no differences in the expression of Akt, p-Akt, p300, and p-p300 between the crush injury rats and the sham-operated rats, indicating that the crush injury itself did not alter the expression of these proteins in the ventral spinal cord (Figures 3A–C). *In vitro*, Res up-regulated the expression of p-Akt and p-p300, which were co-localized in the PC-12 cells (*p*<*0.01*). LY294002, an inhibitor of Akt signaling (Lu et al., 2016), down-regulated the expression of p-p300 (*p*<*0.05*, Figures 3D–F), suggesting that the Res-induced phosphorylation of p300 was mediated via Akt signaling. In addition, these results may indicate that Res enhanced the activity of p300 acetyltransferase, as the phosphorylation of p300 at Ser-1834 is believed to be essential for this activity (Huang and Chen, 2005; Liu et al., 2006; Chen et al., 2015).

### Activation of p300 acetyltransferase mediated res-induced expression of VEGFs in differentiated PC-12 cells

**Figure 4:**
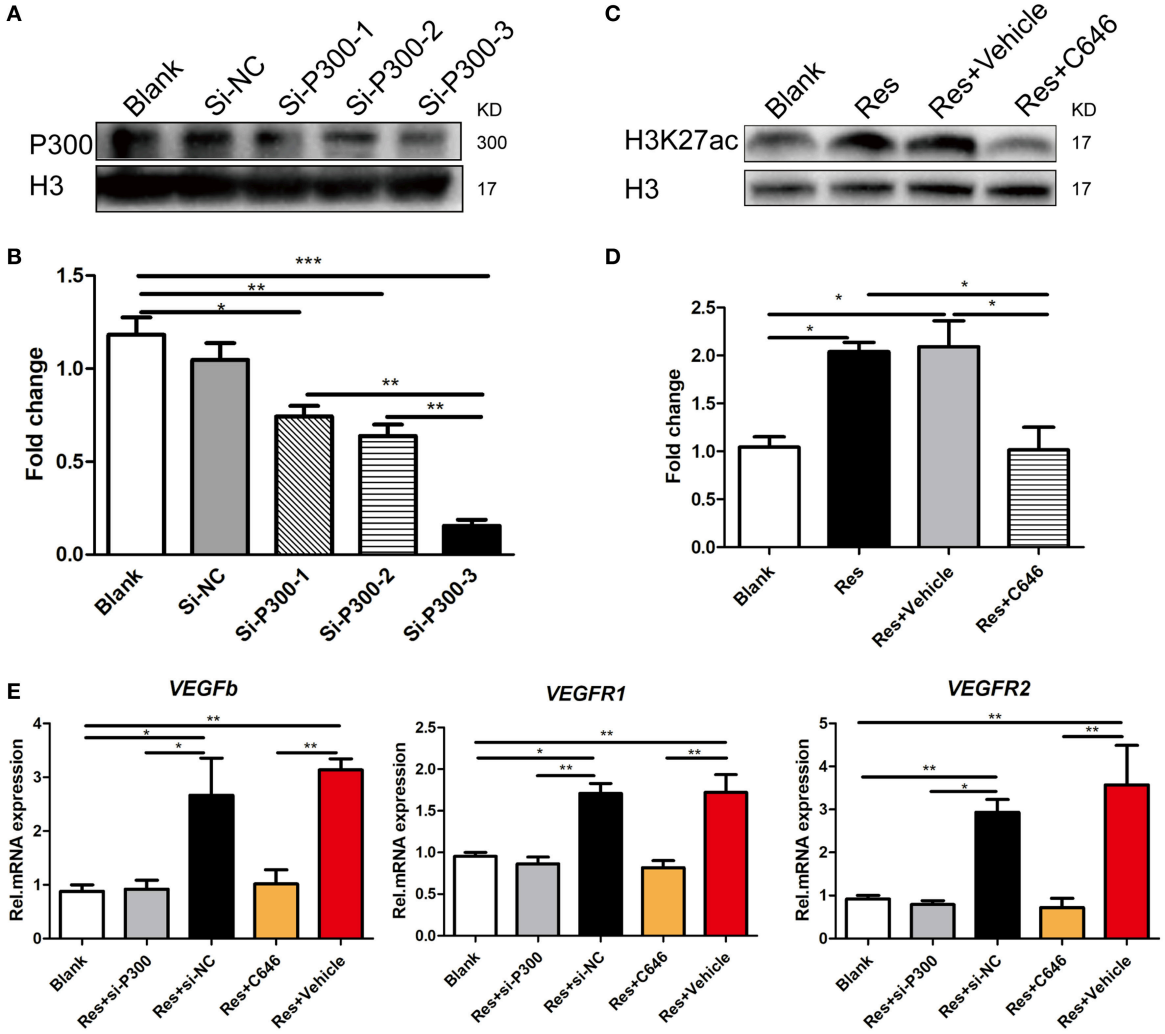
p300 knockdown and the inhibition of p300 acetyltransferase activity separately suppressed the Res-induced expression of VEGFs in differentiated PC-12 cells. **(A,B)** The protein expression of p300 in differentiated PC-12 cells was inhibited by the transfection of three p300-targeting siRNAs. Among these three siRNAs, si-P300-3 showed the highest efficiency of p300 knockdown (^*^P<*0.05*, ^**^P<*0.01*, ^***^P<*0.01*). **(C,D)** Resveratrol (Res) enhanced the expression of H3K27ac, but C646, an inhibitor of p300 acetyltransferase activity, reversed the increase in H3K27ac induced by Res in differentiated PC-12 cells (^*^P<*0.05*). **(E)** si-P300-3 and C646 separately suppressed the Res-induced mRNA expression of VEGFb, VEGFR1, and VEGFR2 in differentiated PC-12 cells (^*^P<*0.05*, ^**^P<*0.01*, ^***^P<*0.01*).

We interrupted p300 acetyltransferase activity by knockdown of p300 via an RNAi assay or by C646, an inhibitor of p300 acetyltransferase activity. After comparing the efficiency of three p300-targeting siRNAs, we found that the best one reduced the p300 expression to 80% of the base level, and thus this siRNA was selected for the experiment (Figures 4A,B). C646 reversed the Res-induced up-regulation of H3k27ac to the basal level (Figures 4C,D). H3K27ac is a well-established target of p300 acetyltransferase (Creyghton et al., 2010; Jin et al., 2011). This result may indicate that C646 effectively blocked the activation of p300 acetyltransferase induced by Res. We then investigated the effect of p300 knockdown and C646 on the expression of VEGFs in differentiated PC-12 cells. Similarly, Res significantly increased the mRNA expression of VEGFa, VEGFR1, and VEGFR2 in PC-12 cells (*P*<*0.01*). Both the p300 knockdown and C646 reversed the expression of these VEGFs to the basal level (Figure 4E). These results suggest that the activation of p300 acetyltransferase mediated Res-induced expression of VEGFs.

### Inhibition of p300 acetyltransferase activity abolished the effect of res on motor repair in crush injury rats

**Figure 5:**
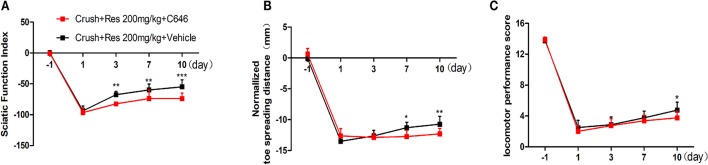
The inhibition of p300 acetyltransferase activity abolished the effect of resveratrol (Res) on motor repair in crush injury rats. The intrathecal treatment of C646 reversed the Res-induced increase of the sciatic nerve index from the third to tenth days after the treatment **(A)**, the normalized toe spreading distance from the seventh to tenth days after the treatment **(B)** and the locomotor performance score on the tenth day after the treatment **(C)** (crush + 200 mg/kg Res vs. crush + 200 mg/kg Res + C646, ^*^P<*0.05*, ^**^P<*0.01*, ^***^P<*0.001, n* = 8 for each group).

We have showed in a previous study that the intrathecal treatment of C646 inhibited p300 acetyltransferase activity on the spinal cord (Zhu et al., 2012). Therefore, we further explored the effect of intrathecal treatment of C646 on Res-induced motor repair. The crush injury rats receiving the concurrent treatment of Res and C646 showed a worse motor function than the crush injury rats which received Res treatment alone. When compared with the Res treated rats, the Res plus C646 treated rats had a decreased SFI (Figure 5A) and normalized toe spreading distance (Figure 5B) on day 7 and day 10 (*P*<*0.05*) after the treatment as well as a decreased locomotor performance score (Figure 5C) on day 10 after the treatment (*P*<*0.05*). These results indicate that the inhibition of p300 acetyltransferase activity by intrathecal C646 treatment abolished the effect of Res on motor repair in the crush injury rats.

## Discussion

Res has been shown to exert its effect in a time- and dose-dependent manner (Stervbo et al., 2006; Bresciani et al., 2014). The doses of Res we used in this study were set according to previous reports in which systematic Res treatment showed benefits on the spinal cord (Ates et al., 2006; Liu et al., 2011). We found Res improved motor function at a dose of both 200 and 50 mg/kg, but the higher dose led to better motor repair than the lower dose. Although the doses of Res we used were higher than those used previously to alleviate the peripheral nerve injury-induced motor deficit, we obtained the motor repair within a period of treatment much shorter than before (Bagriyanik et al., 2014; Tanyeri et al., 2015). For example, in a study performed by Tanyeri et al, it took 2 weeks to observe motor recovery after i.p. treatment with 30 mg/kg Res in a rat model of facial nerve crush injury (Tanyeri et al., 2015). In another study, Bagriyanik et al. found that motor amelioration was identified 2 weeks after i.p. treatment with 10 mg/kg Res in a rat model of sciatic nerve injury (Bagriyanik et al., 2014). In our study, increased SFI was observed as early as 3 days post-injury in rats receiving 200 mg/kg Res, indicating rapid motor repair after the treatment at this dose. Additionally, systematic Res treatment at a dose of 200 mg/kg per day has been considered safe and non-toxic in rats, even when the treatment lasted as long as 90 days (Johnson et al., 2011).

We believe the rapid amelioration of motor function after treatment with 200 mg/kg Res is due to the acceleration of nerve regeneration, as the earlier the nerve repair, the better the functional recovery (Susarla et al., 2007; Gordon et al., 2011). Indeed, peripheral nerve injury not only leads to axon degeneration in the distal portion of the injured nerve, but also triggers an intrinsic growth system and thus induces nerve regeneration from the proximal stump of the injured nerve (Johnson et al., 2011). However, the rate of the intrinsic axon proceeding is sometimes too slow for a functional recovery (Fu and Gordon, 1995; Sulaiman and Gordon, 2000). This is particularly true for impaired motor function, which often has minimal recovery due to the delay in motor axons reaching their target (Hoke et al., 2002; Sakuma et al., 2016). Accelerating nerve regeneration has been shown to promote motor recovery after peripheral nerve injury in rodents (Ma et al., 2011; Tuffaha et al., 2016). In our study, the axons distal to the lesion site were almost degenerated, a state associated with a near complete loss of motor function. Res increased the number of axons in the distal part of the injured nerve, suggesting an acceleration of nerve regeneration which may have contributed to improved motor repair after the crush injury.

The activation of VEGFs in the spinal cord may contribute to the nerve regeneration following Res treatment. Generally, VEGFs are recognized as proangiogenic factors (Shibuya, 2011). Recent studies also showed that they promote nerve regeneration after peripheral nerve injury (Pan et al., 2013; Guaiquil et al., 2014). We found that Res enhanced both mRNA and protein levels of VEGFb, VEGFR1, and VEGFR2 in the spinal cord. In particular, VEGFR1 and VEGFR2 were expressed in the motor neurons of the ventral spinal cord. Res may activate signaling between VEGF and its receptors in the motor neuron and thus promote nerve regeneration. We also showed that Axitinib, a non-specific blocker of VEGFs, inhibited Res-induced neuronal axon growth. These results indicate that the activation of VEGFs, especially VEGFb, VEGFR1, and VEGFR2, contributes to the Res-promoted nerve regeneration observed in rats subjected to sciatic nerve injury.

Res also increased the levels of phosphorylated Akt and phosphorylated p300 at Ser-1834 in the ventral spinal cord. It has been shown that Res activates Akt signaling by enhancing Akt phosphorylation in neurons, leading to a neuroprotective effect (Patel et al., 2011; Zhang et al., 2014). The Akt signaling may then potentiate the acetyltransferase activity of p300 by increasing the level of phosphorylation at Ser-1834 (Huang and Chen, 2005; Liu et al., 2006; Chen et al., 2015). Furthermore, we showed that Res up-regulated the level of H3K27ac, a histone marker associated with the acetyltransferase activity of p300 (Creyghton et al., 2010; Jin et al., 2011). These results together indicate that Res enhanced the acetyltransferase activity of p300. However, previous studies showed that Res is an activator of Sirt1 (Borra et al., 2005), a deacetyltransferase which works in a role contrary to p300 (Kemper et al., 2009). Res has been shown to increase the association between Sirt1 and p300 and thus somehow inhibit the p300-mediated acetylation of the target protein (Kuno et al., 2013; Zhang et al., 2015). However, we did not observe a substantial increase in the expression of Sirt1 mRNA after Res treatment. In fact, sciatic nerve injury caused a dramatic decrease in Sirt1 in the spinal cord, which is consistent with previous findings (Yin et al., 2013; Shao et al., 2014). The effect of Res may not be sufficient to completely reverse the decrease in Sirt1 within such a short period of systematic treatment. Therefore, instead of working as a Sirt1 activator, daily Res treatment for 10 days may, in rat spinal cord affected by sciatic nerve injury, activate p300 acetyltransferase via the Akt signaling pathway.

p300 is a coactivator that links transcriptional complexes including transcription factors and components of basal transcription machinery so as to increase the expression of their target genes (Chan and La Thangue, 2001). The binding of p300 with transcription factors HIF-1-alpha and STAT3 is required for hypoxia-induced transcription of VEGF mRNA (Gray et al., 2005; Kwon et al., 2012; Ding et al., 2017a). Besides its physical interaction with transcriptional complexes, the intrinsic acetyltransferase activity of p300 is also vital for the activation of gene transcription (Ogryzko et al., 1996; Zhu et al., 2014). We found that the Res-induced expression of VEGFs was reversed by either p300 knockdown or by inhibiting p300 acetyltransferase activity. We believe that the activation of p300 acetyltransferase contributed to the Res-induced expression of VEGFs, as the expression of VEGFs was reduced to the basal level either by the inhibition of p300 acetyltransferase activity or by p300 knockdown. We also showed that inhibiting P300 acetyltransferase activity suppressed Res-induced axon growth *in vitro* and motor repair in nerve crush rats. Taken together, these results indicate that Res may enhance transcription of the VEGFs via activating p300 acetyltransferase, and thus promoting nerve regeneration and motor repair after sciatic nerve crush injury.

There are several limitations to this study. First, perhaps due to the short period of treatment, the motor deficit was not totally reversed by Res. Further experiments should be performed to examine whether Res has a better effect on motor repair with a prolonged treatment period. Second, further experiments should be performed on primary cultured neurons to obtain results more reliable than those from the neuronal cell line in the current study. Third, to obtain more direct evidence of the transcriptional regulation of the VEGF genes by p300, experiments such as chromatin immunoprecipitation should be performed to examine the binding of p300 on the promoter or enhancer of these genes.

## Conclusion

Although Res has long been recognized as an activator of Sirt1, in this study we showed that Res activated p300, a protein acetyltransferase which works in a role contrary to Sirt1. The daily systematic Res treatment promoted nerve regeneration and led to rapid motor repair. Res activated p300 acetyltransferase-mediated VEGF signaling in the affected ventral spinal cord, which may have thus contributed to the acceleration of nerve regeneration and motor repair.

## Author contributions

ZD and JC performed experiments, analyzed data, prepared figures, and drafted the manuscript. YS performed experiments, analyzed data. YZ, XY, and WZ performed experiments. QG and CH designed and supervised the experiments, and edited the manuscript. All authors read and approved the final manuscript.

### Conflict of interest statement

The authors declare that the research was conducted in the absence of any commercial or financial relationships that could be construed as a potential conflict of interest.
